# Supplementary material for: VviPLATZ1 is a major factor that controls female flower morphology determination in grapevine
Source: Nat Commun. 2021 Nov 30;12:6995. doi: 10.1038/s41467-021-27259-8 (PMC8632994; doi:10.1038/s41467-021-27259-8)
Supplement: Supplementary file 1 — Supplementary Information [file 41467_2021_27259_MOESM1_ESM.pdf]

VviPLATZ1 is a major factor that controls female flower morphology determination in grapevine

Iocco-Corena *et al.*

Supplementary Table 1. SDR SNP location on chromosome 2

SNP Marker	SNPs identified between PN40024 and Pinot Noir	SNP position in PN40024 [1]	SNP position in CS *f*-haplotype [2]	SNP position in CS H-haplotype [2]	Cabernet Sauvignon *f*-haplotype gene annotation [1]	Locus ID [1]	NCBI SNP Location [3]
VvMT45*	T/A	4237964	4279452	4234550	Sugar phosphate/phosphate translocator	VIT_202s0025g04920	4425548
VvMT50	G/A	4830735	4896680	4830897	Aldolase	VIT_202s0154g00100	Not located [3]
VvMT48	A/T	4832019	4897963	4832178	Aldolase	VIT_202s0154g00100	4889232
VvMT52	C/G	4835442	4901381	4835601	Aldolase	VIT_202s0154g00100	4892655
VvMT55	C/T	4841646	4907578	4841829	Trehalose-phosphate phosphatase (TPP)	VIT_202s0154g00110	4898859
VvMT54	T/C	4841744	4907676	4841917	Trehalose-phosphate phosphatase (TPP)	VIT_202s0154g00110	4898957
VvMT53	A/G	4842302	4908231	4842473	Trehalose-phosphate phosphatase (TPP)	VIT_202s0154g00110	Not located [3]
VvMT61	G/A	4867857	4940251	4886748	KASIII (3-ketoacyl-acyl carrier protein synthase III)	VIT_202s0154g00140	4925070
VvMT136	T/G	4893151	4959576	4946415	VviPLATZ1[4]	VIT_202s0154g00150	Not located [3]
VvMT137	C/A	4893180	4959605	4946444	VviPLATZ1[4]	VIT_202s0154g00150	Not located [3]
VvMT138	A/G	4893222	4959647	4946486	VviPLATZ1[4]	VIT_202s0154g00150	Not located [3]
VvMT66	A/G	4895537	4961978	4948733	FMO1 (FLAVIN-CONTAINING MONOOXYGENASE 1)	VIT_202s0154g00160	4952750
VvMT65	T/C	4895574	4962015	4948770	FMO1 (FLAVIN-CONTAINING MONOOXYGENASE 1)	VIT_202s0154g00160	4952787
VvMT72	G/T	4918002	4983028	4992186	FMO3 (FLAVIN-CONTAINING MONOOXYGENASE 3)	VIT_202s0154g00190	4975215
VvMT70	C/T	4918233	4983259	4992417	FMO3 (FLAVIN-CONTAINING MONOOXYGENASE 3)	VIT_202s0154g00190	4975446
VvMT69	T/A	4920630	4985657	4994813	FMO3 (FLAVIN-CONTAINING MONOOXYGENASE 3)	VIT_202s0154g00190	Not located [3]
VvMT73	G/A	4927393	4992434	5000058	VviFSEX	VIT_202s0154g00200	Not located [3]
VvMT74	C/T	4927524	4992565	5000189	VviFSEX	VIT_202s0154g00200	4984737
VvMT75	G/C	4928268	4993309	5000897	VviFSEX	VIT_202s0154g00200	Not located [3]
VvMT76	C/G	4928304	4993345	5000933	VviFSEX	VIT_202s0154g00200	Not located [3]
VvMT85*	G/T	5037405	5030505	5074644	Phosphatidic acid phosphatase	VIT_202s0154g00230	4980192

[1] From http://www.grapegenomics.com. PN40024_Chr02_V2.1.

[2] From http://www.grapegenomics.com. Cabernet Sauvignon (CS) *H*- and *f*-haplotypes (permenant repository: https://doi.org/10.5281/zenodo.3827985).

[3] From NCBI, https://www.ncbi.nlm.nih.gov/. *Vitis vinifera* cultivar PN40024 chromosome 2, 12X, whole genome shotgun sequence, Assembly 12X (GCF_000003745.3), Chr2 (NC_012008.3).

[4] This article.

*SNPs located outside SDR

Supplementary Table 2. Sequenom primers for SNP genotyping

SNP Marker	PCR Primer 1 [1]	PCR Primer 2 [1]	Extension Primer [2]	*f/f* [3]	*H/f* [3]	*H/H* [3]
VvMT48	acgttggatgAAGAGGCAGCAGAATTTGGG	acgttggatgGACTCCCTTTGGTTAATCTC	cTCATTTTTGCTATTGTATTGCCTC	A/A	A/T	T/T
VvMT52	acgttggatgGGAGTTCATACCAGAAATGC	acgttggatgAAAGAAGCCACAGTTTTCCC	CACAGTTTTCCCTTCCCCA	C/C	C/G	G/G
VvMT55	acgttggatgTATTGGTTAGTCCTGCGCAC	acgttggatgTTTCTCCCTGAACACCCTTG	TTGGTACATAATCTCTGGCT	C/C	C/T	T/T
VvMT54	acgttggatgTTTCTCCCTGAACACCCTTG	acgttggatgTATTGGTTAGTCCTGCGCAC	ctACCTCTAAAACCTAAGCATGG	T/T	T/C	C/C
VvMT53	acgttggatgAAAATTCACTGGCAGGCTGG	acgttggatgCCACGACTCATGTACATACC	CTGTGGCAGGGTAAG	G/G	G/A	A/A
VvMT61	acgttggatgGGGAACATCCAGTACTGAAC	acgttggatgGCCCTTACCTGCTCTTTATG	CAGGCACTTAAATGCTTC	A/A	A/G	G/G
VvMT136	acgttggatgGCTGATGGATTGTTCTCTTG	acgttggatgTGAGAAGATCGGACGGAAAG	gggacGCGCTCTGCAACTCA	G/G	G/T	T/T
VvMT137	acgttggatgTGAGAAGATCGGACGGAAAG	acgttggatgGCTGATGGATTGTTCTCTTG	cctaGCTGGCCTGCACTGC	A/A	A/C	C/C
VvMT138	acgttggatgTGAGAAGATCGGACGGAAAG	acgttggatgGCTGATGGATTGTTCTCTTG	agctCAGCAGCGTCTCTGT	G/G	G/A	A/A
VvMT66	acgttggatgTCATTCCCAGTTGTGTGACC	acgttggatgAACAAAGGAAAGCCCTGGAG	CAAAGCTGGTGGAAAAA	G/G	G/A	A/A
VvMT65	acgttggatgAACAAAGGAAAGCCCTGGAG	acgttggatgTCATTCCCAGTTGTGTGACC	ATACCAATGGCATCGTAAC	C/C	C/T	T/T
VvMT72	acgttggatgGTAGTGCTTTGGTGTGTCAG	acgttggatgCAAATGGATAGCAGGTGCTC	TTCAGCCTTTTATTTGTCAC	T/T	T/G	G/G
VvMT70	acgttggatgAAGCTGATCAAACCAAGAGG	acgttggatgTTTCCACCACATTTGGCTCC	GGCTTTCCTTTGTTGGT	T/T	T/C	C/C
VvMT69	acgttggatgCCTTGAGATAAAGCGAGACC	acgttggatgTTTTCTGGACTACCCCTTCG	aGTATCCACTGGTGAACC	A/A	A/T	T/T
VvMT73	acgttggatgGGTCCATATCGATGCTACTG	acgttggatgTTCTTTCAGGTGGGCTCTAC	CCAAGGTGTTTGCTCC	A/A	A/G	G/G
VvMT74	acgttggatgTTCTTTCAGGTGGGCTCTAC	acgttggatgCCCTTCTTGCTAGTATCCTC	cAGAAAAGAACTCTTATCACCTAA	T/T	T/C	C/C
VvMT75	acgttggatgGGAAGTGTGTCGGAATTTTC	acgttggatgTCAACAGCAAGCTCTTCAGG	TCAGTTTGAGGAAGGTG	C/C	C/G	G/G
VvMT76	acgttggatgGGAAGTGTGTCGGAATTTTC	acgttggatgTCAACAGCAAGCTCTTCAGG	ggGCTGGAGAAAACCAAATG	G/G	G/C	C/C
VvMT85	acgttggatgAAGCAAGGAAGGGAAGAAGG	acgttggatgAACTCTAGAGGAATGGCCAC	GTGAGATGCATGCCCTTATTGTAGA	T/T	T/G	G/G

[1] PCR amplification primers for the sequenom MassARRAY assay. Uppercase represents grape-specific sequences and lowercase bases are non-grape sequences used for multiplex PCR.

[2] Single base extension primer for the sequenom MassARRAY assay. Uppercase represents grape-specific sequences and lowercase bases are non-grape sequences used for multiplex genotyping.

[3] Homozygous female (f/f), heterozygous hermaphrodite (H/f), homozygous hermapharodite (H/H). SNP genotypes were called on either the + or - DNA strand depending on the SNP marker.

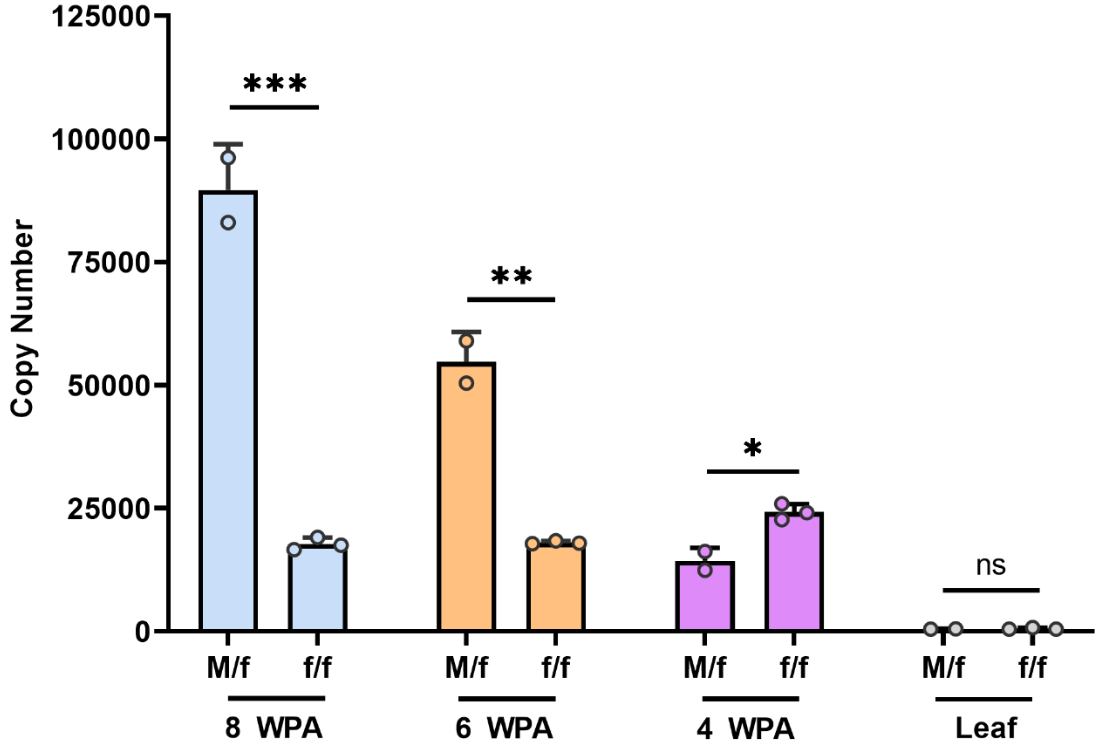

Supplementary Fig. 1. *VviPLATZ1* expression in male flowers. Transcript levels determined for *VviPLATZ1* in 03C003V0060 (*M/f*) and 04C023V0003 (*f/f*) microvine flowers at eight and six weeks prior to anthesis (WPA) and leaves. Eight WPA is marked by stamen development and pistil initiation[2]. Filament elongation and ovule development occurred at six WPA[1]. Data are presented as mean values +/- SD. Significant differences determined by two-tailed Student's *t*-test indicated by asterisks, *p = 0.013, **p = 0.0014, ***p = 0.00073 (n = 2 for *M/f* at all time points (8, 6 and 4 WPA, as well as leaf; n = 3 for *f/f* at all time points. (8, 6 and 4 WPA, as well as leaf). Note: the parentage of the 03C003V0060 (*M/f*) microvine is 00C001V0008 x Richter 110. Based on the parentage of Richter 110 (*V. berlandieri* cv. Boutin B x *V. rupestris* cv. du Lot), the *M*-factor is derived from *V. rupestris* cv. du Lot[2]. The 00C001V0008 microvine was derived from the selfing (S1) of L1 Pinot Meunier[3]. Source data are provided as a Source Data file.

```
            04C023V0006(H):CAAAAGAGTATAAAAATGAAAATGGTG
     Cabernet Sauvignon(H):CAAAAGAGTATAAAAATGAAAATGGTG
                 Merlot(H):CAAAAGAGTATAAAAATGAAAATGGTG
               Semillon(H):CAAAAGAGTATAAAAATGAAAATGGTG
              Zinfandel(H):CAAAAGAGTATAAAAATGAAAATGGTG
               Riesling(H):CAAAAGAGTATAAAAATGAAAATGGTG
             Chardonnay(H):CAAAAGAGTATAAAAATGAAAATGGTG
         Cabernet Franc(H):CAAAAGAGTATAAAAATGAAAATGGTG
   Black Corinth Seedless(H):CAAAAGAGTATAAAAATGAAAATGGTG
            03C003V0060(H):CAAAAGAGTATAAAAATGAAAATGGTG
VITVvi_sDVIT3603.16_v1.1(M):CAAAAGAGTATAAAAATGAAAATGGTG
            V. arizonica(M):CAAAAGAGTATAAAAATGAAAATGGTG
            04C023V0003(f):CACAAGAGTAAAAAAAAAGAAAATGGT
     Cabernet Sauvignon(f):CACAAGAGTAAAAAAAAAGAAAATGGT
                 Merlot(f):CACAAGAGTAAAAAAAAAGAAAATGGT
               Semillon(f):CACAAGAGTAAAAAAAAAGAAAATGGT
              Zinfandel(f):CACAAGAGTAAAAAAAAAGAAAATGGT
         Cabernet Franc(f):CACAAGAGTAAAAAAAGAAAATGGTG
   Black Corinth Seedless(f):CACAAGAGTAAAAAAAGAAAATGGTG
     VITVvi_sDVIT3603.07(f):CACAAGAGTAAAAAAAGAAAATGGTG
          VITVvi_s034-16(f):CACAAGAGTAAAAAAAGAAAATGGTG
VITVvi_sDVIT3603.16_v1.1(f):CACAAGAGTAAAAAAAGAAAATGGTG
            V. arizonica(f):CACAAGAGTAAAAAAGTGAAAATGGTG
```

Supplementary Fig. 2. Conserved DNA polymorphisms in the 5’ UTR and coding sequences of female *VviPLATZ1* alleles. Nucleotide sequence alignment of the 5’ UTR and coding sequence of *VviPLATZ1* alleles from 04C023V0006 (*H/H*) and 04C023V0003 (*f/f*), as well as sequenced hermaphrodite, male and female alleles derived from selected domesticated and wild *Vitis* species at grapegenomics.com. *VviPLATZ1* alleles were aligned and compared using the CLC Genomics Workbench 21.0.3 (Qiagen Bioinformatics) multiple sequence alignment function.

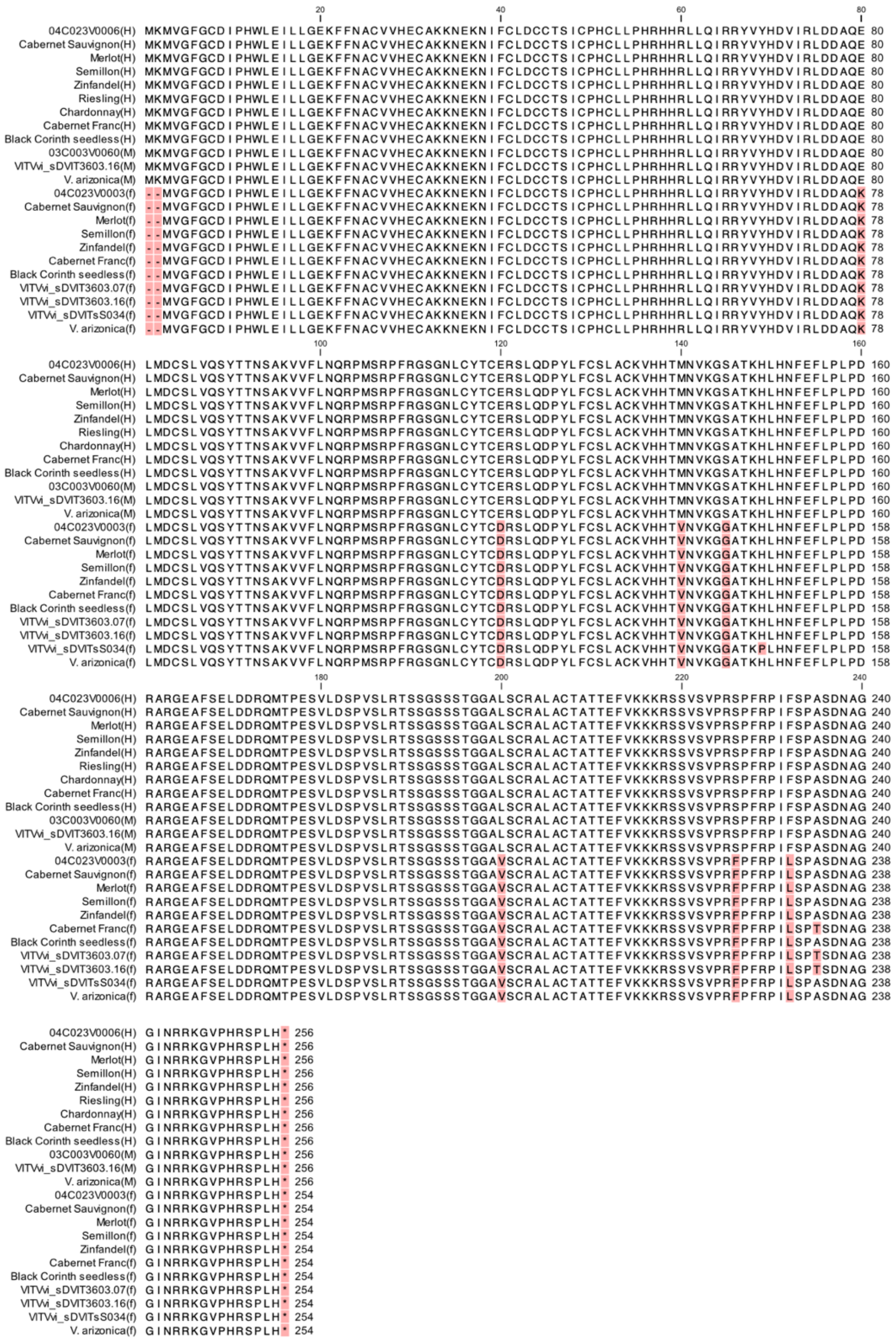

Supplementary Fig. 3. Conserved DNA polymorphisms in female *VviPLATZ1* alleles alter the protein sequence. Amino acid sequence alignment derived from hermaphrodite, male and female VviPLATZ1 alleles. VviPLATZ1 proteins sequences from selected domesticated and wild *Vitis* species included in the alignment were derived from grapegenomics.com). VviPLATZ1 sequences derived from *Vitis* hermaphrodite, female and male alleles were aligned and compared using the CLC Genomics Workbench 21.0.3 (Qiagen Bioinformatics) multiple sequence alignment function.

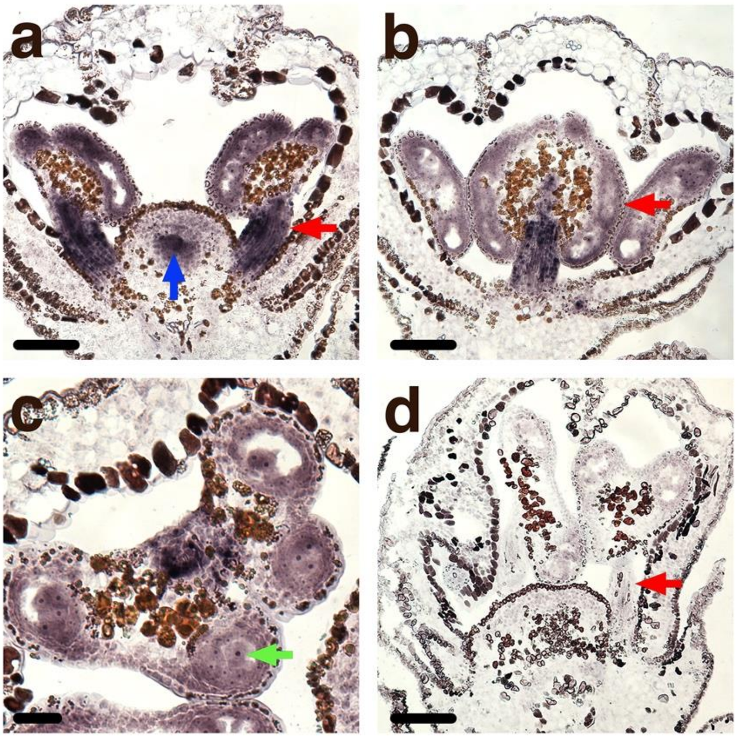

Supplementary Fig. 4. Expression of *VviPLATZ1* in male flowers at 6 WPA of development. a, b *VviPLATZ1* mRNA was located in stamens (red arrow), including the filaments and anthers in the male (*M/f*) microvine 03C003V0060. The blue arrow points at *VviPLATZ1* expression in the ovule. **c** In the anthers, *VviPLATZ1* expression was detected in microspores (green arrow) and the surrounding tapetum cells. **d** *VviPLATZ1* mRNA levels were low in 04C023V0003 flowers. Red arrow points at stamens consisting of filaments and anthers. The representative images were derived from three biological replicas for 03C003V0060 and 04C023V0003. Scale bar is 100 µM for panels **a**, **b** and **d**. For panel **c**, scale bar is 25 µM.

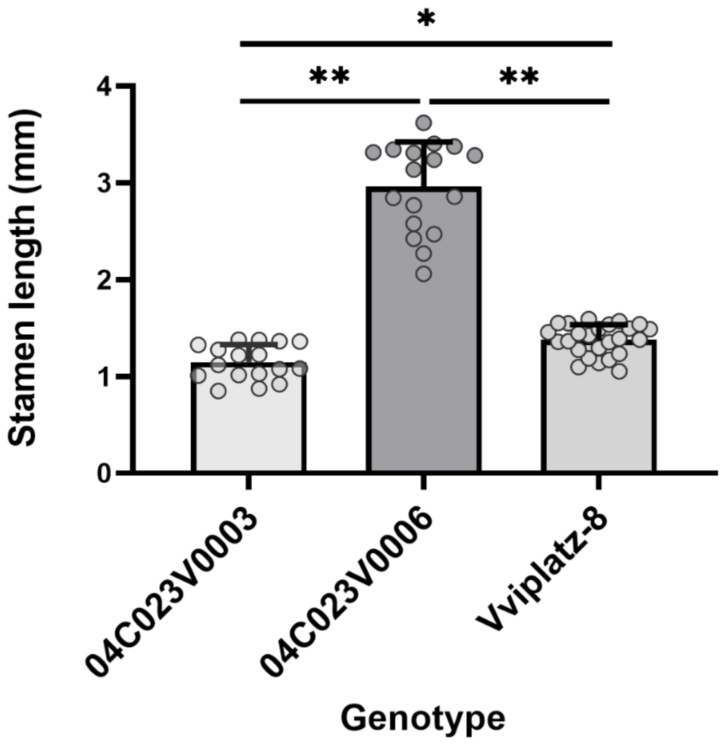

Supplementary Fig. 5. Stunted stamens produced in *Vviplatz1-8* are similar in length to stamens initiated in 04C023V0003 (*f/f*) compared to 04C023V0006 (*H/H*). The average length of stunted (non-reflexed) stamens produced in 04C023V0003 (*f/f*) and *Vviplatz1-8* were 1.2 and 1.4 mm, respectively ($n_{04C023V003}$ = 17, SD = 0.18; $n_{Vviplatz1\text{-}8}$ = 25, SD = 0.16). The average length of stamens produced in 04C023V0006 was 3.0 ($n_{04C023V0006}$ = 17, SD = 0.46), which was significantly longer than stunted stamens (non-reflexed) initiated in 04C023V0003 and *Vviplatz-8*. Analysis of variance followed by Tukey honestly significant difference showed that the stunted stamens (non-reflex) initiated in *Vviplatz1-8* and 04C023V0003 were more similar in length (*p = 0.035) than 04C023V0006 (**$p = 7.3 \times 10^{-12}$). SD, standard deviation. Source data are provided as a Source Data file.

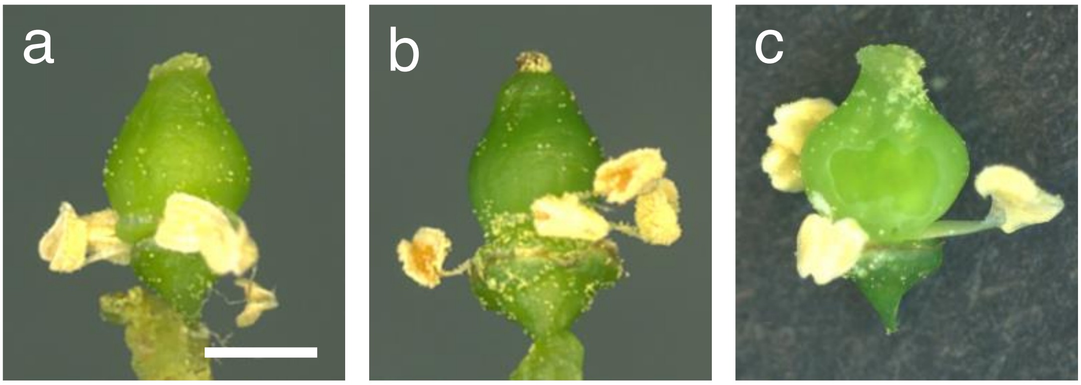

Supplementary Fig. 6 Flower morphology of CRISPR/Cas9 gene edited *Vviplatz1-1/2, Vviplatz1-4* and *Vviplatz1-6* plants. Representative images of flowers from **a** *Vviplatz1-1/2*, **b** *Vviplatz1-4* and **c** *Vviplatz1-6* plants. The scale bar is 1.0 mm and is representative for all flower images (**a-c**).

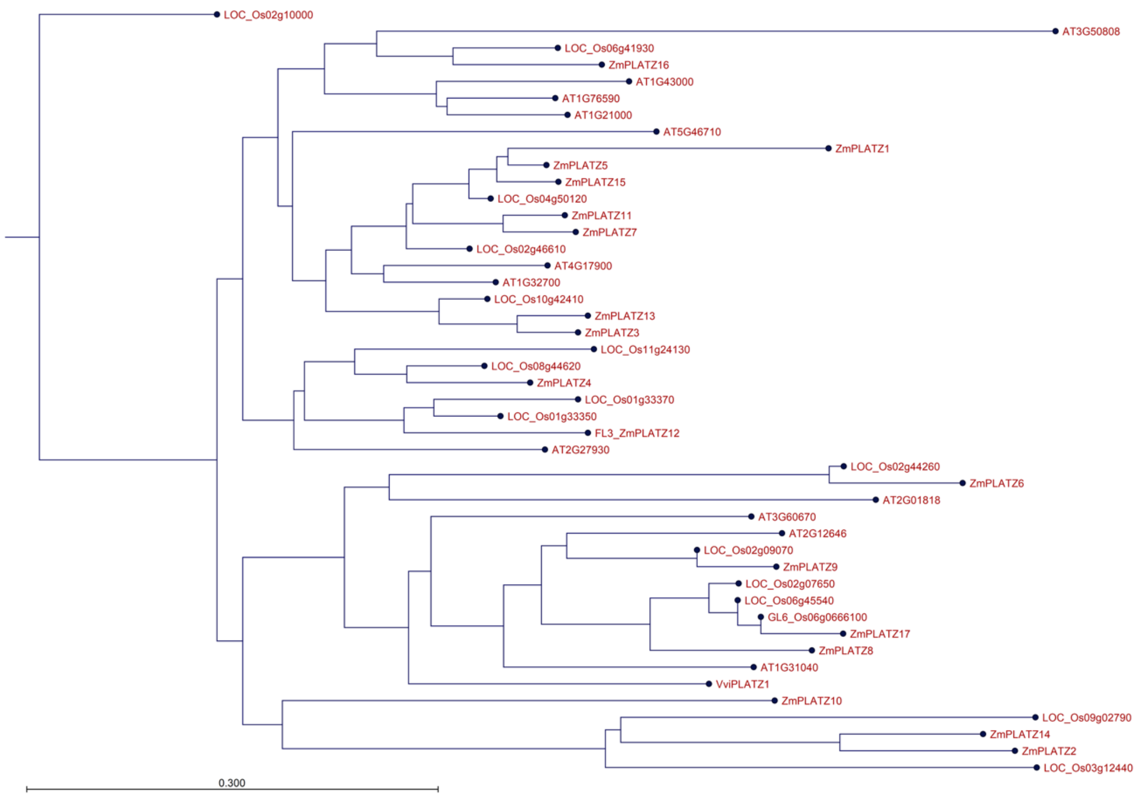

Supplementary Fig. 7. Phylogenetic analysis of VviPLATZ1 and related PLATZ proteins from *Zea mays*, *Oryza sativa* and *Arabidopsis thaliana*. The maximum likelihood phylogeny in the CLC genomics workbench was used to construct the phylogenetic tree from PLATZ proteins sequences. Scale bar represents evolutionary distances.

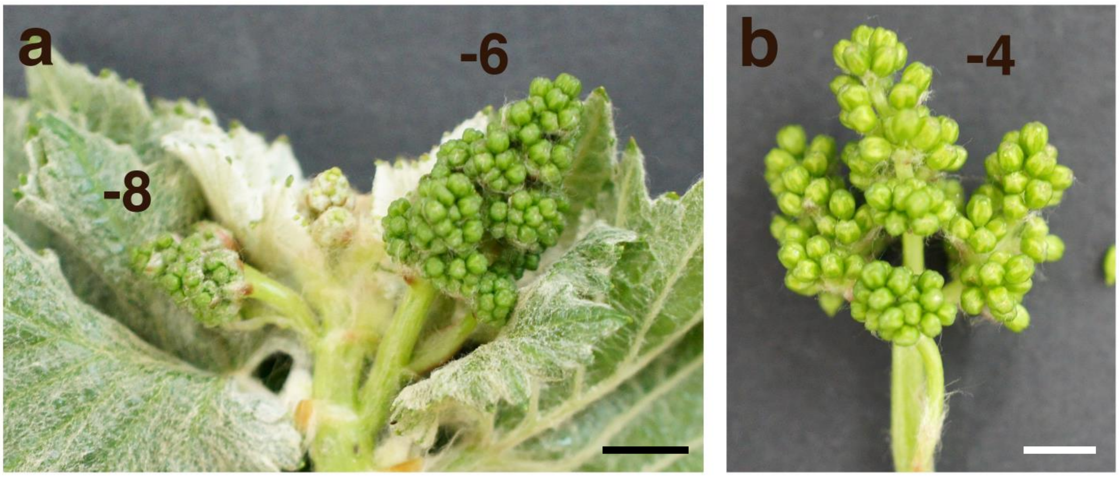

Supplementary Fig. 8. Inflorescences with flowers at developmental stages 8, 6 and 4 weeks prior to anthesis (WPA). **a** Image of shoot with inflorescences with flowers at eight and six WPA. **b** Image of inflorescence with flowers at four WPA RNA was isolated from flowers dissected from inflorescences at 8, 6 and 4 WOA for RT-qPCR. The stages of flower development were previously described[1]. The scale bars are 5.0 mm.

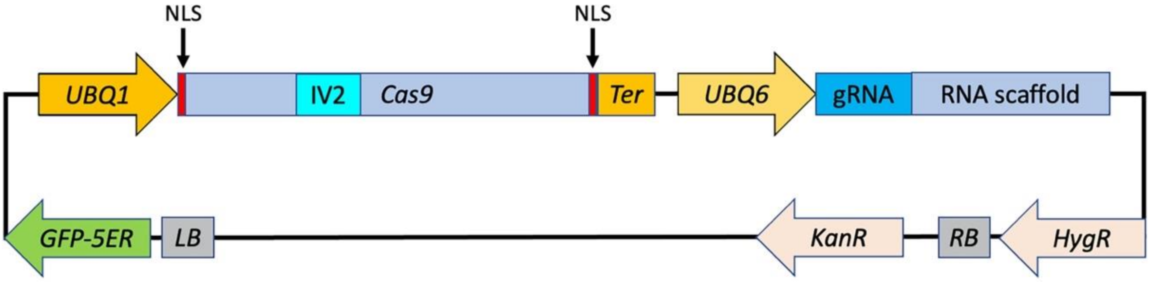

Supplementary Fig. 9. Diagram of CRISPR/Cas9 vector used to edit *VviPLATZ1*. The Cas9-sgRNA cassette was synthesized. Arabidopsis *UBIQUITIN1* (*UBQ1*) and *UBQ6* were used to drive expression of *Cas9* and sgRNAs, respectively. The *UBQ1* terminator (Ter) was used to end transcription of *Cas9*. The approximate position of nuclear localization sequences and the potato *IV2* intron are indicated by red and light blue boxes, respectively. The *Agrobacterium* selection marker is kanamycin resistance (KanR). Transgenic plants are selected with an endoplasmic reticulum localized green fluorescent protein (GFP-5ER) and hygromycin resistance (HygR). The right and left T-DNA borders, RB and LB, respectively, are shown in grey boxes. The binary vector containing the FS1 guide RNA (gRNA) is referred to as pVCAS9FS1, while pVCAS9FS4 contains the FS4 guide RNA (gRNA).

Supplementary references

1. Poupin, M. J. *et al.* in *The flowering process and its control in plants: gene expression and hormone interaction* (ed M. W. Yaish) Ch. 8, 173-197 (Research Signpost, 2011).

2. Riaz, S. *et al.* Genetic diversity and parentage analysis of grape rootstocks. *Theor Appl Genet* **132**, 1847–1860 (2019).

3. Chaïb, J. *et al.* The grape microvine – a model system for rapid forward and reverse genetics of grapevines. *Plant J* **62**, 1083-1092 (2010).
