# Supplementary material for: VviPLATZ1 is a major factor that controls female flower morphology determination in grapevine
Source: Nat Commun. 2021 Nov 30;12:6995. doi: 10.1038/s41467-021-27259-8 (PMC8632994; doi:10.1038/s41467-021-27259-8)
Supplement: Supplementary file 3 — Description of Additional Supplementary Files [file 41467_2021_27259_MOESM3_ESM.pdf]

Description of Additional Supplementary Files

Supplementary Data 1
Description: Genotyped plants with *SDR* SNP set
